# NLRP3 Inflammasome: A New Perspective on Revealing Hotspots and Trends in Uric Acid Metabolism Disorders through Bibliometric Analysis

**DOI:** 10.2174/0118715303376897250702230058

**Published:** 2025-07-22

**Authors:** Yi Xu, Yu Wang, Chengjin Lu, Zeyu Liu, Zhijian Lin, Xiaomeng Zhang, Bing Zhang

**Affiliations:** 1 School of Chinese Materia Medica, Beijing University of Chinese Medicine, Beijing 102488, China

**Keywords:** NLRP3 inflammasome, uric acid metabolism disorders, gout, uric acid nephropathy, hyperuricemia, hotspots, frontiers

## Abstract

Uric acid metabolism disorders have become major health concerns, primarily causing conditions such as hyperuricemia, uric acid nephropathy, and gout. Recent research has focused on these disorders’ underlying pathological mechanisms and pharmacological treatments, emphasizing the role of the NLRP3 inflammasome. This study aims to analyze and summarize the current research status, hotspots, and trends in this field from a new perspective of the NLRP3 inflammasome, providing insights for future research and disease treatment. Based on the Web of Science Core Collection (WoSCC), this study systematically retrieved all publications related to the NLRP3 inflammasome and uric acid metabolism disorders published between 2009 and 2023. A comprehensive statistical analysis and visualization of the gathered data were conducted using VOSviewer, CiteSpace, R-bibliometrix, Microsoft Excel, Scimago Graphica, and Pajek. A total of 585 articles from 250 journals spanning 49 countries/regions worldwide were identified, demonstrating a consistent annual increase in publications. China contributed the largest number of publications, followed by the United States and South Korea. FRONTIERS IN IMMUNOLOGY emerged as the most prolific journal, while ANNALS OF THE RHEUMATIC DISEASES boasted the highest impact factor. The current research hotspots can be broadly categorized into three themes: the molecular mechanisms underlying NLRP3 inflammasome activation, the pathological mechanisms of NLRP3 inflammasome involvement in uric acid metabolism disorders, and the development of therapeutic compounds/ drugs targeting the NLRP3 inflammasome. Furthermore, the safety of drugs targeting NLRP3 inflammasome may emerge as a significant research trend in the future. Over the past 15 years, the crucial role of the NLRP3 inflammasome in uric acid metabolism disorders has attracted considerable attention. In the foreseeable future, the role of the NLRP3 inflammasome in uric acid metabolism disorders will continue to be a research focus. The development and safety of drugs targeting the NLRP3 inflammasome will remain a future research trend.

## 1 INTRODUCTION

Uric acid metabolism disorders arise when the delicate balance between uric acid production and excretion is disrupted, leading to a spectrum of conditions, including hyperuricemia, uric acid nephropathy (UN), and gouty arthritis. Hyperuricemia, marked by abnormally elevated blood uric acid levels due to increased production and/or decreased excretion, stands as the second most common metabolic disorder in China after diabetes [1]. Uric acid nephropathy, also known as gouty nephropathy, stems from the accumulation of monosodium urate (MSU) crystals in the distal collecting tubules and medullary interstitium, which are intimately linked to inflammatory responses [2]. Gouty arthritis, a form of crystal-induced arthropathy, occurs from the deposition of urate crystals in the joints and is closely tied to hyperuricemia [3]. The shared underlying pathology of these three diseases is a uric acid metabolism disorder, hinting at a common pathological mechanism.

The NOD-, LRR-, and pyrin domain-containing protein 3 (NLRP3) inflammasome is an intracellular multimeric protein complex composed of the NLRP3 protein, apoptosis-associated speck-like protein (ASC), and pro-cysteinyl aspartate-specific proteinase-1 (pro-caspase-1) [3]. This NLRP3 inflammasome is a pivotal “molecular processing platform” for producing the inflammatory cytokine IL-1β. Extensive research has highlighted the crucial role of the NLRP3 inflammasome and its associated pathways in uric acid metabolism disorders. Clinical studies have demonstrated significantly elevated serum IL-1β levels in patients with asymptomatic hyperuricemia, uric acid nephropathy, or gout, accompanied by alterations in the NLRP3/IL-1β inflammatory signaling pathway [3, 4-7]. Further pathological mechanistic studies have revealed that the NLRP3 inflammasome is activated upon exposure to soluble uric acid and MSU crystals. This activation triggers the release of inflammatory cytokines IL-1β and IL-18, induces pyroptosis, and ultimately leads to inflammation in the kidneys, joints, or systemic tissues [8-11]. Currently, drugs targeting the NLRP3 inflammasome for the treatment of uric acid metabolism disorders are under development. However, the existing research on the NLRP3 inflammasome and uric acid metabolism disorders remains fragmented. Therefore, a systematic review and analysis of the existing studies, research hotspots, and future trends in this field are imperative.

Unlike previous studies that primarily focused on the NLRP3 inflammasome in individual inflammatory diseases, this study highlights its evolving research hotspots in uric acid metabolism disorders, particularly its disease-specific mechanisms and targeted therapeutic interventions. Our objective is to trace the progression, identify key research hotspots, and forecast future trends in this field, offering valuable insights for investigations into pathological mechanisms and novel therapeutic approaches for uric acid metabolism disorders.

## 2 METHODS

### 2.1 Data Collection

The data for this study were obtained from the Web of Science Core Collection (WoSCC), which is one of the most widely accessed academic databases and comprehensively covers research records on the NLRP3 inflammasome in the context of uric acid metabolism disorders [12, 13]. Therefore, the WoSCC was chosen as the target database. The literature search was conducted on June 1, 2024. The selection criteria for the search terms were based on existing literature and the Medical Subject Headings (MeSH).

Before the formal search, a preliminary search was conducted, investigating the NLRP3 inflammasome in relation to hyperuricemia, uric acid nephropathy, and gouty arthritis individually. The results indicated that searching for articles on the association between each disease and the NLRP3 inflammasome was limited, potentially affecting the stability and representativeness of our findings [14, 15]. Given the shared pathological basis of hyperuricemia, uric acid nephropathy, and gouty arthritis, we combined these conditions with the NLRP3 inflammasome in our search strategy. This approach also aligned with the prevailing research trend in this field. The search strategy followed references [13, 16], optimizing the combination of search terms to ensure the accuracy and reliability of the results. The official search strategy is as follows: TS = ((NLR Family, Pyrin Domain Containing 3 Protein) OR (NACHT, LRR and PYD Domains Containing Protein 3) OR (NLRP3 inflammasome) OR (Nod-like receptor protein 3 inflammasome)) AND TS=((hyperuricemia) OR (uric acid nephropathy) OR (hyperuricemic nephropathy) OR (Gouty Arthritis) OR (Arthritis, Gouty) OR (Gouty Arthritis) OR (gout) OR (Gout Arthritis) OR (Arthritis, Gout)).

**Fig. (1):**
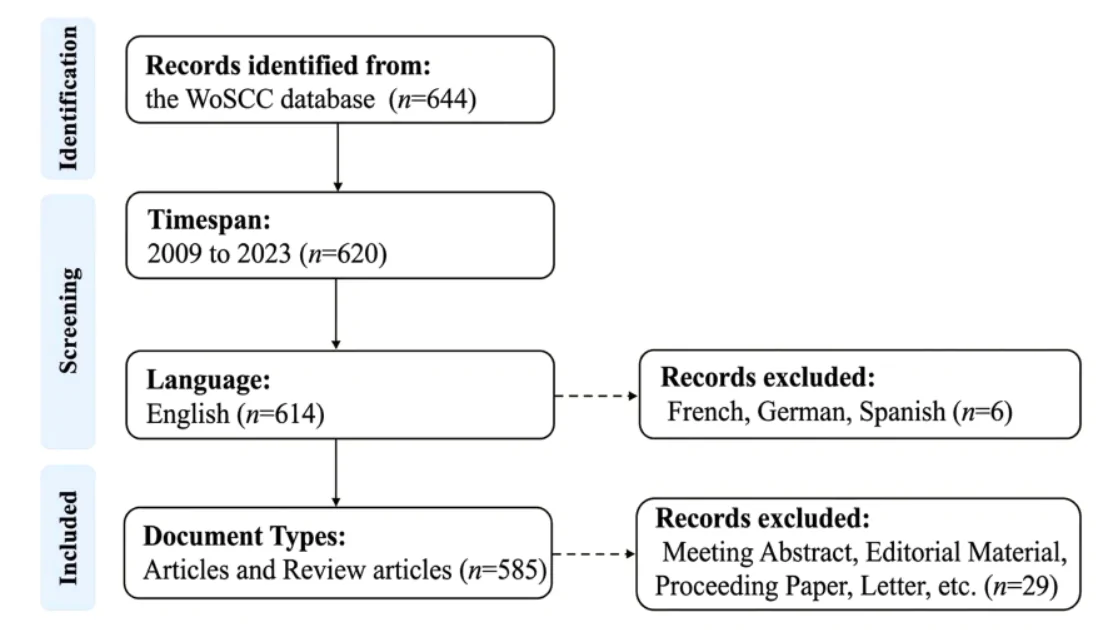
Literature Search Flowchart (Initial search: 644 records; Time range screening (2009-2023):24 records were excluded, leaving 620 articles; Language screening (in English): 6 articles were excluded, leaving 614 articles; Document type screening (articles and reviews):29 articles were excluded, and 585 articles were ultimately included.).

Additionally, our initial search revealed that the earliest records in the WoSCC on the NLRP3 inflammasome and uric acid metabolism disorders dated back to 2009. Proceedings papers, meeting abstracts, and editorial materials are excluded because they are considered knowledge still being developed and may duplicate or overlap when later converted into articles [13]. Therefore, the search period was set from 2009 to 2023, focusing solely on original English-language articles and reviews. The literature search process is shown in (Fig. **1**). A total of 644 records were initially retrieved from the WoSCC. After applying the time range filter (2009–2023), 24 records were excluded. Additionally, 6 non-English records were removed based on language criteria. Finally, 29 irrelevant document types (*e.g.*, meeting abstracts, editorial materials) were excluded, retaining only articles and reviews. This resulted in 585 records for analysis.

### 2.2 Data Analysis

The 585 records were exported from the WoSCC in plain text format. Data analysis and visualization were performed using various tools: the analysis module of WOSCC, VOSviewer (1.6.20.0), CiteSpace (6.3.1.0), R-bibliometrix, Microsoft Excel (2021), Scimago Graphica (1.0.42.0), and Pajek (5.18).

This study utilized a multidimensional analysis approach, including temporal evolution, geographical distribution, institutional collaboration networks, and keyword co-occurrence analysis, to comprehensively reveal research trends on the NLRP3 inflammasome in uric acid metabolism disorders. In conjunction with Microsoft Excel, we analyzed annual publications, annual citations, institutional publications, author publications, journal publications, research categories, and funding agencies. VOSviewer was employed to examine institutional collaboration, author collaboration, and keyword clustering. Scimago was used to cluster countries/regions and visualize geographic distributions. R-bibliometrix was utilized to visualize national collaborative publications, author annual publications, and journal annual publications, and to conduct topic analyses. Finally, CiteSpace was used to perform keyword emergence analysis, create a keyword timeline, and conduct co-citation analyses.

## 3 RESULTS

### 3.1 Global Overview

A total of 585 papers, comprising 439 articles and 146 review articles, published between January 2009 and December 2023, were included in this study. These papers were published across 250 journals by 3335 authors from 52 countries/regions. The total number of citations received by these papers was 30991, with an H-index of 87. This information is illustrated in Fig. (**2**).

### 3.2 Publication Outputs Trend

As depicted in Fig. (**3**), the cumulative number of publications in the field of NLRP3 inflammasomes and uric acid metabolism disorders showed an overall increasing trend from 2009 to 2023, despite a brief decline. The number of publications peaked in 2022, with 103 papers. Similarly, the number of citations has increased each year, reaching a peak of 5927 in 2023. This indicates that the field is thriving, with growing interest among researchers in exploring the relationship between NLRP3 inflammasomes and uric acid metabolism disorders.

Notably, the growth during 2015–2016 may be attributed to breakthroughs in understanding the role of the NLRP3 inflammasome in uric acid-related metabolic diseases, particularly driven by the publication of high-impact studies (*e.g.*, references [17, 18]). This spurred a surge in research activity. The slight decline in 2020–2021 is likely due to the COVID-19 pandemic, as many research teams redirected their focus to pandemic-related studies. The peak in 2022–2023 may reflect significant progress in elucidating the molecular mechanisms of the NLRP3 inflammasome in uric acid metabolism disorders, mainly through key experimental studies (*e.g.*, reference [19]), which significantly boosted citation rates in the field.

### 3.3 Cooperation and Distribution of Countries/Regions

A total of 49 countries contributed to these 585 publications. Table **1** lists the top 10 most active countries in terms of the number of publications, with China leading the field with 313 papers (53.50% of the total), 8713 citations, and an H-index of 46. The USA has the greatest influence in this field, evidenced by its highest H-index and number of citations. Other notable contributors include Korea, Germany, and the United Kingdom.

**Fig. (4):**
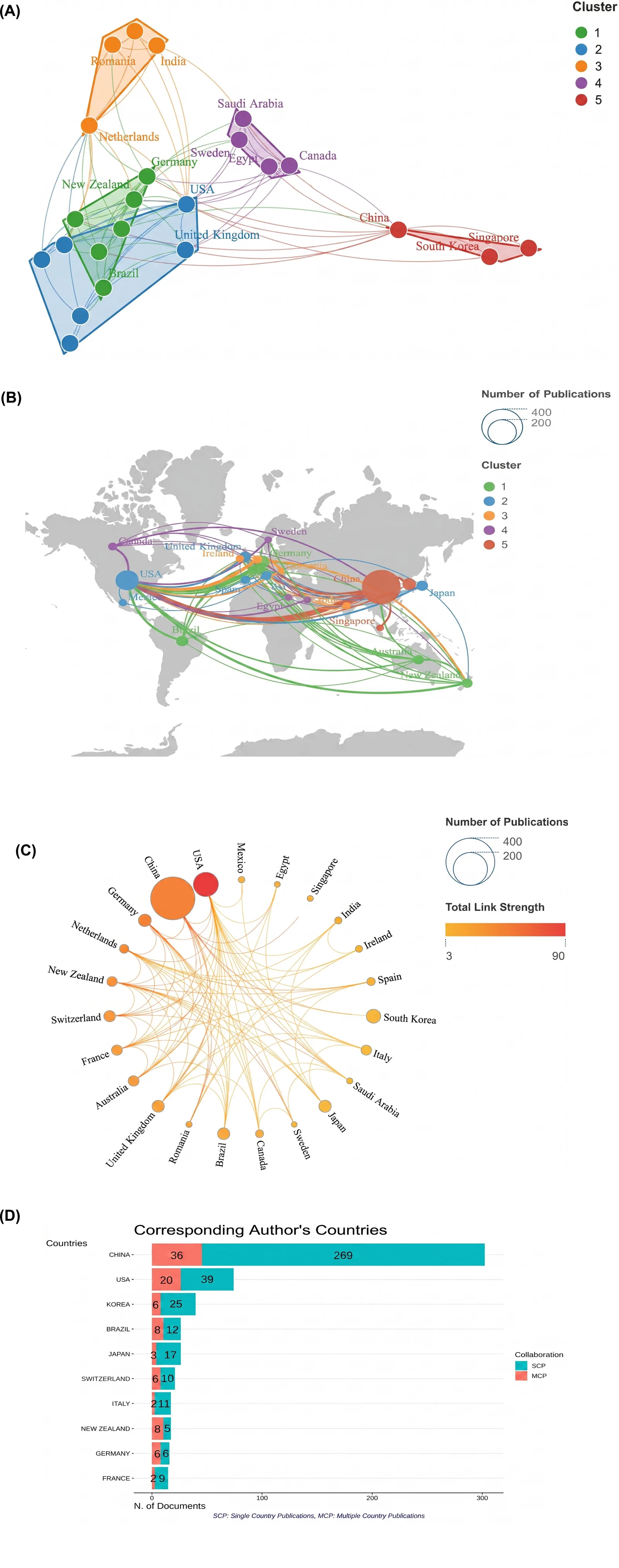
Analysis of country/region cooperation. **(A)** Cluster diagram of country/region cooperation (threshold is the number of publications greater than five). 23 countries form five cooperative clusters, with the same color representing closer cooperation. **(B)** Geographical map of country/region cooperation. **(C)** Chord diagram of national cooperation. The larger the circle, the more publications. The redder the color, the stronger the cooperation. **(D)** Histogram of the top 10 contributing countries. Green bars represent publications from a single country/region, while pink bars represent collaborative publications involving multiple countries/regions.

Twenty-three countries had published more than five papers. (Figs. **4A** and **4B**) illustrate the strength of national collaborations, categorized into five clusters based on activity levels. Collaboration intensity is closely tied to geographical proximity, as seen in the strong Asian partnerships (*e.g.*, China with South Korea and Singapore). (Fig. **4C**) highlights that the United States exhibits the most substantial international collaborations, likely due to its economic strength and extensive research resources. Fig. (**4D**) shows that China leads in single-country publications with 269 articles, followed by the United States, reflecting China's significant focus on this field, potentially driven by the rising prevalence of uric acid-related metabolic diseases [20, 21]. However, international collaboration remains limited, with China, the United States, and Brazil ranking as the top three contributors. This suggests that current research on the NLRP3 inflammasome and uric acid metabolism disorders primarily focuses on domestic collaboration, with substantial potential for strengthening international partnerships.

### 3.4 Analysis of Affiliations

Table **2** lists the top ten institutions actively studying NLRP3 inflammasomes and uric acid metabolism disorders. Notably, 80% of these institutions are located in China, with the remaining two being the University of Lausanne in Switzerland and the Universidade de São Paulo in Brazil. This indicates that China has conducted extensive research in this field. Fig. (**5**) shows the clustering network of institutional cooperation, which is divided into eight clusters based on the intensity of institutional cooperation. The same color symbolizes closer cooperation, and the more connections there are, the greater the level of cooperation, such as between the University of Otago and the University of Lausanne.

### 3.5 Author's Contribution

**Fig. (6):**
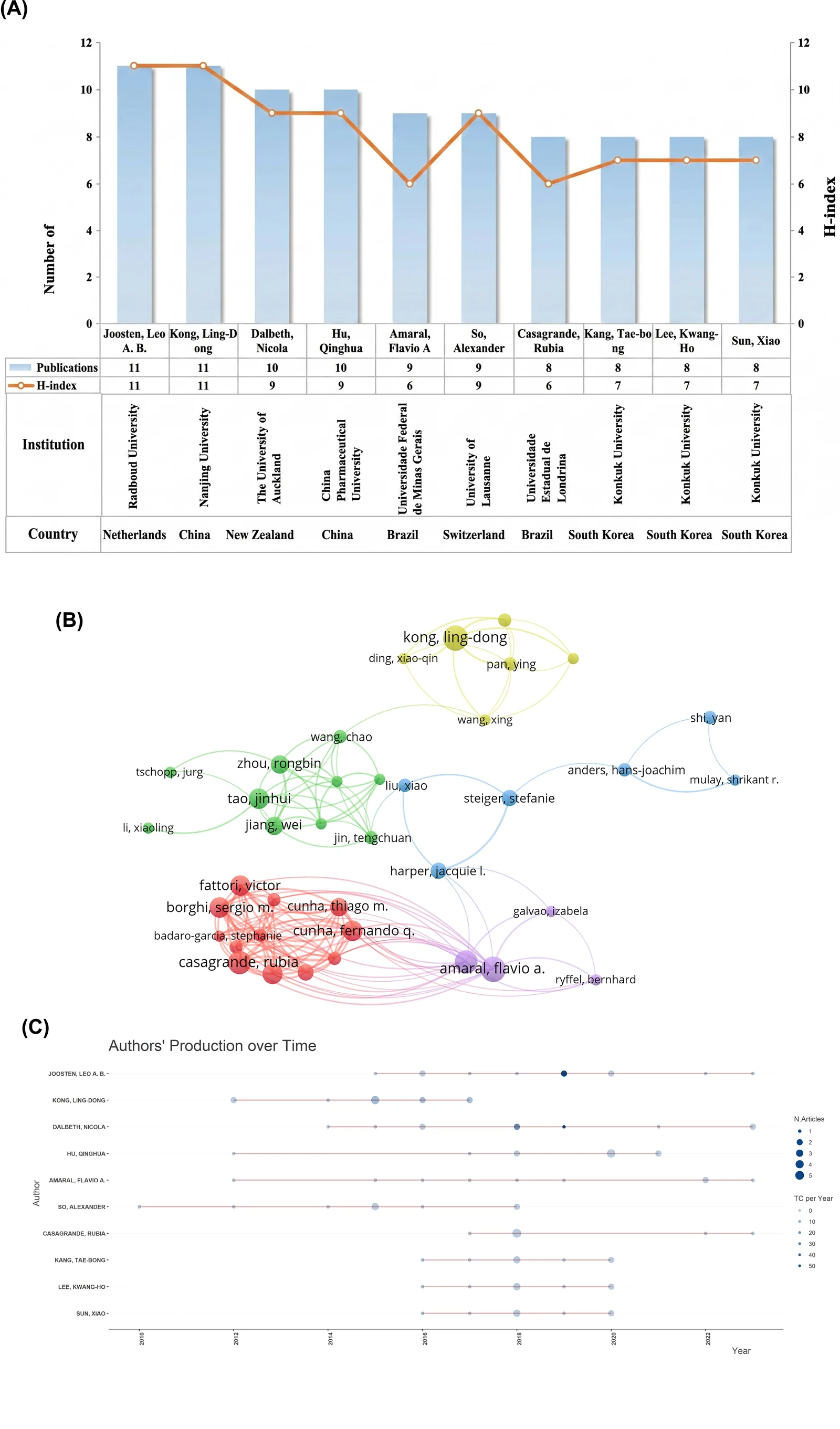
Analysis of author collaboration. **(A)** Top 10 authors with the most publications and their H-indices. The blue bars represent the number of publications, and the white nodes represent the H-index. **(B)** Cluster graph of authors with more than three publications. The size of the nodes indicates the number of publications by the authors, the same color indicates the presence of more active collaboration, and the number and thickness of the node connections are positively related to the intensity of collaboration. **(C)** Annual output of the top ten authors in terms of publications. The darker the color of a node, the more citations it receives, whereas the larger the node, the more publications it has.

A total of 3335 researchers have contributed to 585 articles on NLRP3 inflammasomes and uric acid metabolism disorders over the past 15 years. (Fig. **6A**) depicts the top ten most productive authors, who collectively authored 84 articles, accounting for 14.36% of all articles. Joosten and Leo A.B. from Radboud University and Kong and Ling-Dong from Nanjing University were the most prolific authors, with 11 articles and the highest H-index. Fig. (**6B**) shows the top 10 authors in terms of publications over time, with Amaral, Flavio A. exhibiting the most extended research cycle in the field, spanning from 2012 to 2023. The node size represents the number of publications, and the total number of publications by the top 10 authors peaked in 2018, suggesting a heightened level of research activity on NLRP3 inflammasomes and uric acid metabolism disorders during that year. Darker node color represents more citations, with Joosten, Leo A.B., ranked first in citations, followed by Dalbeth, Nicola, both in the year 2019. (Fig. **6C**) presents a collaborative clustering analysis of authors based on the number of publications exceeding five. The study reveals varying degrees of direct or indirect collaboration between authors, with the red clusters represented by Casagrande and Rubia demonstrating more positive internal and external cooperation.

### 3.6 Academic Journals

**Fig. (7):**
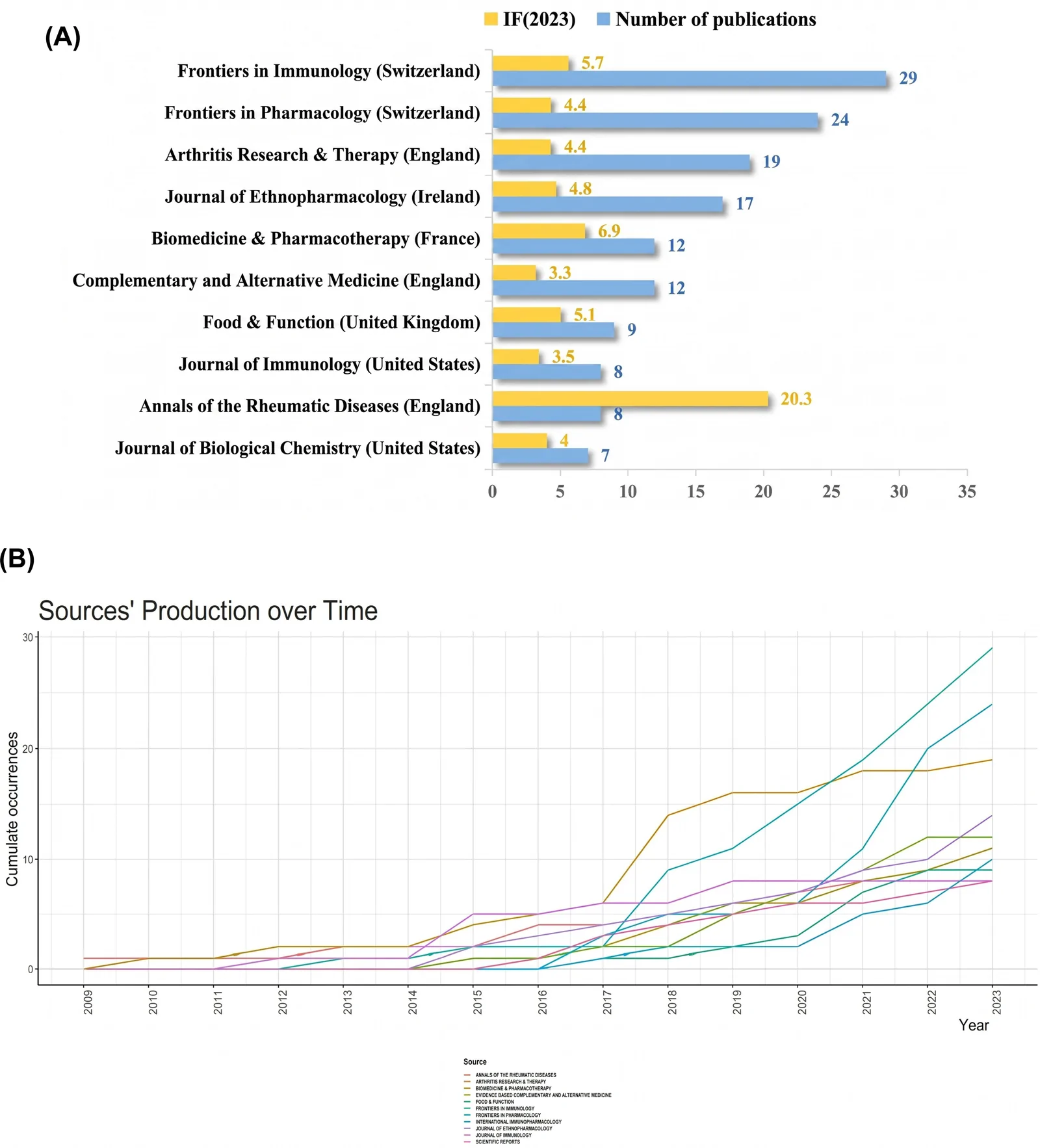
Analysis of journal publications. **(A)** Top 10 academic journals by publication volume. **(B)** Trends in the annual output of the top 10 academic journals.

A total of 250 academic journals have published 585 articles on NLRP3 inflammasomes and uric acid metabolism disorders. (Fig. **7A**) displays the top ten journals in terms of publication volume, focusing on pharmacology, immunology, and joint disease-related journals, providing a preferred reference for selecting journals for submission. *Frontiers in Immunology* has the highest number of publications since 2009 (n=29), followed by *Frontiers in Pharmacology* (n=24) and *Arthritis Research & Therapy* (n=19). All journals except *Complementary and Alternative Medicine* had an impact factor of three or higher, with *Annals of the Rheumatic Diseases* having the highest impact factor (IF=20.3). (Fig. **7B**) indicates that the number of journal publications has increased over time, with *Frontiers in Immunology*, *Arthritis Research & Therapy*, and *Frontiers in Pharmacology* showing the most significant growth, suggesting a focus on NLRP3 inflammasomes and uric acid metabolism disorders.

### 3.7 Research Category and Funding Agencies

There are 51 research categories involved in studies on NLRP3 inflammasomes and uric acid metabolism disorders. Fig. (**8A**) displays the top ten research categories in this field, with pharmacology (n=141) and immunology (n=105) having the highest frequency of occurrence, followed by biomedical molecular biology, rheumatology, and food science and technology. In terms of financial support, 867 institutions provided funding. Fig. (**8B**) shows the top ten funding organizations, with the National Natural Science Foundation of China (NSFC) providing the most support (n=179), accounting for 20.06% of the total support institutions. This indicates that the NSFC places great importance to research in the field of NLRP3 inflammasomes and uric acid metabolism diseases and has made significant contributions.

### 3.8 Keywords Hotspots

Keywords are the essence of research content, enabling us to swiftly understand the research direction and hotspots. In this study, 585 publications contained 2238 keywords, with 184 appearing more than five times. VOSviewer grouped these keywords into four clusters based on their degree of association, as depicted in Fig. (**9A**). The red cluster primarily focuses on the structure and function of the NLRP3 inflammasome. The green cluster centers on association studies of the NLRP3 inflammasome and metabolic diseases, such as hyperuricemia. The blue cluster examines the pathological mechanism of NLRP3 inflammasome in gout, particularly MSU-induced gouty attacks. The yellow cluster emphasizes clinical studies of NLRP3 inflammasome in drugs, specifically drug safety. While research encompasses both hyperuricemia and gouty arthritis, studies on gouty arthritis and the NLRP3 inflammasome are more prevalent, aligning with current research trends, as illustrated in the density map in Fig. (**9B**).

Keyword burst analysis aids in tracking research hotspots over specific periods. As shown in Fig. (**9C**), research hotspots can be categorized into three phases: 2009-2014, 2012-2019, and 2019-2023. From 2009 to 2014, the focus was on the molecular mechanisms of NLRP3 inflammasome activation, with keywords like “IL-1beta”, “caspase-1 activation”, and “NF-kappa b”. Between 2012 and 2019, research hotspots expanded to include the study of the NLRP3 inflammasome and metabolic diseases, such as “gout arthritis”, “kidney inflammation”, and “insulin resistance”. This period also witnessed the development of disease models, indicated by the keyword “model”. From 2019 to 2023, the focus shifted to the relationship between the NLRP3 inflammasome and hyperuricemia, as well as kidney damage, reflected in keywords like “serum uric acid”, “xanthine oxidase”, and “renal injury”. Additionally, studies on drug efficacy and safety, such as “febuxostat” and “safety,” became prominent.

Thematic maps were utilized to analyze and visualize the evolution of a topics. As shown in Fig. (**9D**), the horizontal axis of the thematic map represents centrality, while the vertical axis represents density. The first quadrant (top right) comprises motor themes, representing important and well-developed themes. For instance, the involvement of NLRP3 inflammasomes in the pathogenesis of gouty arthritis and the development of related animal models. The second quadrant (top left) indicates highly developed but isolated themes, which are established but not closely linked to current research hotspots. Examples include C-reactive protein and familial Mediterranean fever. The third quadrant (bottom left) contains emerging or declining themes, also known as fringe themes, indicating themes in their nascent or declining stages of research, with weak connections to other themes and requiring further investigation. Examples include double-blind, efficacy, and osteoarthritis. The fourth quadrant (bottom right) comprises basic themes that may emerge as future research hotspots and trends. Examples include the relationship between NLRP3 inflammasomes and hyperuricemia, as well as urate nephropathy.

### 3.9 Analysis of Co-citation

Co-citations unveil research topics that are closely tied to specific fields. Fig. (**10A**) depicts the top ten highly cited references. Duewell *et al*. [22] exhibited the highest intensity of research, revealing that cholesterol crystals induce phagolysosomal damage by activating the NLRP3 inflammasome in macrophages, subsequently triggering the development of atherosclerotic inflammation. Zhou RB *et al*. [23] explored the physiological role of the NLRP3 inflammasome, emphasizing that it can sense mitochondrial dysfunction, producing ROS, and further activate the NLRP3 inflammasome, illustrating the close relationship between mitochondrial damage and inflammatory diseases.

The timeline view of keywords illustrates the evolving patterns of research hotspots over time. As shown in Fig. (**10B**), there are nine clusters: NLRP3 inflammasome, ASC oligomerization, NF-kappa b signal pathway, monosodium urate, gouty inflammation, gouty arthritis, oxidative stress, coronary artery disease, rheumatic disease, and atherogenesis. The structures and activation mechanisms of the NLRP3 inflammasome have been the primary research focus from 2009 to 2023. Clusters # 4 and # 5 show that research on gouty arthritis was most intensive in 2012, whereas “safety” began to gain attention in 2023. Little research has been conducted on the correlation between coronary artery disease, rheumatic disease, and atherogenesis.

## 4 DISCUSSION

### 4.1 General Trends

This study presents the first bibliometric analysis of global research on NLRP3 inflammasomes and uric acid metabolism disorders. The cumulative number of publications in this field remains relatively low, exhibiting significant annual growth fluctuations, which underscores the need for further investigation. The publication peak was observed in 2022. China tops the list in terms of the number of publications, with eight of the top ten institutions located there and providing substantial funding, indicating China's prominent research activity in this field. The United States, however, leads in citations and H-index, suggesting a higher overall article quality. *Frontiers in Immunology* and *Frontiers in Pharmacology* have published the highest number of articles in this field, while *Annals of the Rheumatic Diseases* boasts the highest IF. Keyword clustering and topic analysis reveal a concentration on the activation mechanisms of NLRP3 inflammasomes, as well as the pathological and pharmacological studies of NLRP3 inflammasomes in gout and UN. Since 2012, research on NLRP3 inflammasomes and hyperuricemia has progressively garnered attention. Notably, in 2023, safety emerged as a prominent topic of interest.

### 4.2 Hotspots and Frontiers

From the perspective of keyword clustering, thematic analysis, and timeline distribution, current research hotspots concentrate on three aspects: the activation mechanisms of the NLRP3 inflammasome, the pathological roles of the NLRP3 inflammasome in uric acid metabolism disorders, and drug research targeting the NLRP3 inflammasome as a therapeutic target for uric acid metabolism disorders. Additionally, the safety of NLRP3 inflammasome-related drugs is likely to emerge as a future research trend.

#### 4.2.1 Activation Mechanisms of NLRP3 Inflammasome Dominate Research Focus

The NLRP3 inflammasome was first proposed by Martinon *et al*. [24] in 2002. NLRP3 inflammasomes are predominantly expressed in immune cells such as macrophages and neutrophils, as well as in various tissues and organs, including renal tubular endothelial and epithelial cells. The pathophysiological mechanisms of the NLRP3 inflammasome are a focal point of research, encompassing studies on its function, activation pathways, and signal transduction. These studies form the foundation for understanding the pathological mechanisms underlying uric acid metabolism disorders.

In its resting state, the NLRP3 inflammasome exists in a self-inhibited form, characterized by extremely low expression levels of its component proteins. Upon stimulation, it assembles into a cytosolic complex in which activated caspase-1 cleaves pro-IL-1β and pro-IL-18 into their mature forms, IL-1β and IL-18, respectively. These mature cytokines then initiate an inflammatory response [10].

Activating the NLRP3 inflammasome necessitates priming and activation signals [25]. The priming signal involves the activation of the NF-κB pathway by endogenous cytokines (*e.g.*, IL-1β and tumor necrosis factor) or pathogen-associated molecular patterns (such as LPS), which upregulate the transcriptional expression of NLRP3and pro-IL-1β/IL-18 and facilitate the maturation of caspase-1 [26]. The activation signal encompasses a wide range of stimuli, including bacteria, viruses, ATP, or crystalline substances (*e.g.*, uric acid crystals, silica, cholesterol crystals), that activate NLRP3 and promote its assembly with NIMA-related kinase 7 (NEK7), ASC, and caspase-1 into the NLRP3 inflammasome complex, thereby activating caspase-1 [25].

NLRP3 activation involves several upstream transduction signals, such as reactive oxygen species (ROS) generation, K+/CI efflux, Ca+ influx, and lysosomal dysfunction [10]. Among them, the role of ROS in activating the NLRP3 inflammasome has garnered significant attention, as evidenced by the distribution of keywords and subject terms. These transduction signals intertwine and interact with each other. Further elucidation is required to determine whether NLRP3 inflammasome activation results from the parallel or independent actions of these signals, and whether a common mechanism exists among these signal transductions.

Recently, discoveries have revealed that NEK7 is a core component of NLRP3, which, through its interaction with NLRP3, disrupts the inactive state and transforms NLRP3 into the active NLRP3 inflammasome disc [20, 27, 28]. Studies have found that uric acid can induce tubular epithelial cell injury by activating the NEK7/NLRP3 signaling pathway [15]. However, several questions remain unanswered: How does NEK7 perceive uric acid stimulation? Can NEK7 also sense MSU stimulation? And is NEK7 a potential therapeutic target? These issues necessitate further research.

#### 4.2.2 Role of NLRP3 Inflammasome Activation In Uric Acid Metabolism Disorders

Investigating uric acid metabolism disorders from the perspective of NLRP3 inflammasome represents both a current research hotspot and future trend. Soluble uric acid or MSU crystals can trigger the production of IL-1β and other inflammatory cytokines by directly or indirectly activating the NLRP3 inflammasome. This activation leads to a cascade of inflammatory responses, which in turn exacerbate uric acid metabolism disorders. Consequently, this section will delve into the mechanisms by which the NLRP3 inflammasome is implicated in the pathological processes of hyperuricemia, uric acid nephropathy, and gout. We aim to unravel the intricate interplay between NLRP3 inflammasome activation and uric acid metabolism disorders.

#### 4.2.3 Activation of the NLRP3 Inflammasome may be a Potential Mechanism for Hyperuricemia

Hyperuricemia is recognized as a chronic inflammatory disease that is intimately linked to the NLRP3 inflammasome [29-32]. Clinical research has shown that asymptomatic hyperuricemia patients display elevated levels of serum IL-1β and alterations in the NLRP3/IL-1β signaling pathways compared to healthy individuals [4-6]. These findings suggest a potential role for the NLRP3 inflammasome in the development and progression of hyperuricemia. However, the relationship between hyperuricemia and the NLRP3 inflammasome remains unclear.

Certain researchers believe that soluble uric acid, similar to MSU, functions as an endogenous damage-associated molecular pattern that promotes the assembly of NLRP3 and ASC in macrophages, leading to NLRP3 inflammasome activation and increased IL-1β production [33]. This mechanism may involve uric acid-induced mitochondrial oxidative stress, which triggers mitochondrial membrane depolarization [34]. Additionally, soluble uric acid can activate NLRP3 and induce IL-1β production through other pathways, such as NF-κB and TXNIP [35, 36].

On the other hand, some studies suggest that inflammation induced by NLRP3 inflammasome activation can cause tubular damage, which may impair the kidney's ability to filter uric acid and exacerbate hyperuricemia. Research has indicated that administering NLRP3 inhibitors to hyperuricemic rats can significantly reduce their serum uric acid levels [37].

While these findings provide valuable insights, further exploration is needed to fully understand the mechanisms underlying the interaction between soluble uric acid and the NLRP3 inflammasome, as well as to clarify the potential therapeutic implications of targeting this pathway in hyperuricemia.

#### 4.2.4 Activation of NLRP3 Inflammasome Accelerates the Progression of UN

Uric acid nephropathy is an inflammatory condition resulting from the retention of soluble uric acid or the deposition of urate crystals in the tubules and interstitium [38, 39]. The primary clinical manifestations of UN encompass kidney stones formed by crystals, chronic interstitial nephritis, and renal fibrosis. Among the multiple factors contributing to UN progression, the activation of the NLRP3 inflammasome by soluble uric acid and urate crystals, which triggers an inflammatory response, stands out as the most prominent.

On the one hand, uric acid and MSU effectively stimulate the NLRP3 inflammasome in various renal cell types, thereby initiating renal inflammation. For example, soluble uric acid and MSU can activate the NLRP3 inflammasome in renal proximal tubular cells, facilitating IL-1β maturation and inducing pyroptosis and autophagy [13]. In addition, podocytes, mesangial cells, renal neutrophils, and macrophages can all be stimulated by uric acid or MSU, directly or indirectly activating the NLRP3 inflammasome and releasing inflammatory factors [40-43].

On the other hand, NLRP3 inflammasomes activated by uric acid or MSU interact with multiple signaling pathways, inducing renal inflammation and accelerating tubulointerstitial fibrosis, thereby exacerbating the progression of UN. Regarding renal inflammation, NLRP3 inflammasomes synergize with the NF-κB signaling pathway. Soluble uric acid and MSU regulate the NF-κB signaling pathway in proximal tubular epithelial cells (PTECs) through the TLR4 receptor, subsequently activating the NLRP3/caspase-1/IL-1β axis and triggering renal inflammation [11, 44]. Activated NF-κB promotes NLRP3 transcription, forming a vicious cycle of NLRP3 inflammasome activation that exacerbates renal injury [45]. Intriguingly, activated NLRP3 inflammasomes can also trigger the adiponectin-adiponectin receptor 1 signaling pathway, thereby reducing local inflammation and renal injury [46], suggesting potential negative regulatory interactions among these pathways. In renal tubulointerstitial fibrosis, the NLRP3 inflammasome works synergistically with the TGF-β/Smads signaling pathway to regulate the production and degradation of the extracellular matrix and promote epithelial-mesenchymal transition, inducing myofibroblast formation and further exacerbating renal fibrosis [47, 48]. Additionally, UN involves signaling pathways such as ERK1/2, mTOR, MAPK, PhoA/ROCK, and PI3K/Akt, which directly or indirectly interact with the NLRP3 inflammasomes, collectively driving UN progression [49].

In conclusion, further study of the similarities and nuances of NLRP3 inflammasome activation in different renal cell types is crucial. NLRP3 inflammasomes collaborate with multiple signaling pathways in the development of UN. Future research should investigate the role of NLRP3 inflammasomes in various signaling pathways to determine whether they present potential therapeutic targets for UN.

#### 4.2.5 The core Pathological Mechanism behind NLRP3 Inflammasome-mediated Gout Attacks

The study of the NLRP3 inflammasome's role in gout has consistently garnered significant attention in the research community. Gout, an inflammatory disease, arises due to the deposition of MSU in joints (such as the ankle, knee, elbow, wrist, and fingers) or other connective tissues, such as ear cartilage [50]. The activation of the NLRP3 inflammasome stands as the pivotal pathological process during gout attacks, involving mechanisms including K^+^ efflux [51], Ca^2+^ signaling [52], lysosome rupture [53], mitochondrial dysfunction, and ROS generation [54]. Once activated, the NLRP3 inflammasome not only promotes the maturation and secretion of IL-1β and IL-18, but also induces synovial cells and other immune cells to produce inflammatory factors such as prostaglandin E2 and IL-6, thereby further enhancing the inflammatory response [55, 56].

Despite the relatively mature research stage on the NLRP3 inflammasome in gout, several areas still require refinement. One critical aspect is the development of more suitable animal models for gouty arthritis. The most classic modeling method currently involves the direct injection of an MSU suspension into the ankle joints of rats or mice, mimicking the typical inflammatory response seen in clinical gout attacks, including joint swelling, fever, and pain [57]. Studies have demonstrated that in rats and mice with acute gouty arthritis, there is a significant elevation in the expression of NLRP3, ASC, and caspase-1 in synovial tissue, accompanied by increased serum IL-1β levels [58, 59].

Poultry, notably chickens and quails, have emerged as advantageous animal models for gouty arthritis due to their similar uric acid metabolic pathways to humans [60]. Our research team has developed a gout model using quails, which showed notably elevated uric acid and inflammation levels. MSU crystals were visible in the synovial fluid, and the pathogenic mechanism was closely linked to ROS release and NLRP3 inflammasome activation [19, 61, 62]. During the same period, our group also created a rat model of urate renal deposition, providing an essential experimental platform for studying uric acid metabolism disorders [63]. However, the quail model has limitations, primarily due to the scarcity of commercially available reagents specific to quail species indicators, such as ASC and caspase-1 antibodies. This limitation hampers in-depth pathological or pharmacodynamic studies. Therefore, there is an urgent need to develop reagents tailored to the quail species. In summary, establishing an optimal gout model remains a significant challenge that requires immediate attention in gout research.

#### 4.2.6 Research and Prospects of NLRP3 Inflammasome as a Therapeutic Target

The keyword and topic analysis reveals that drug therapy for uric acid metabolism disorders currently constitutes a focal point of research endeavors. Considering the pivotal role of the NLRP3 inflammasome in such disorders, therapies targeting this inflammasome possess promising developmental advantages. The ongoing research on NLRP3 inflammasome inhibitors is extensive, encompassing clinical trial drugs, drug repositioning strategies, synthetic compounds, natural products, and traditional Chinese medicines (Tables **3** and **4**).

#### 4.2.7 Investigating the Therapeutic Effect of Clinical Trial Drugs Targeting NLRP3 Inflammasome

Among the clinical trial reports on NLRP3 inhibitors for treating uric acid metabolism disorders, only three drugs have been identified with indications for gout. Dapansutrile, a novel β-sulfonyl nitrile compound with oral activity, inhibits NLRP3 oligomerization and IL-1β secretion by disrupting the ASC-caspase-1 interaction [64]. In Phase II clinical trials, dapansutrile demonstrated favorable safety and efficacy in alleviating joint pain and inflammation in gout patients [65].

Furthermore, allopurinol, a frontline therapeutic agent for hyperuricemia, has been shown to inhibit NLRP3 activation, thereby mitigating uric acid-induced renal inflammation [66]. However, it is noteworthy that most NLRP3 inflammasome inhibitors are still in the clinical trial phase. With the gradual release of clinical data, the development of NLRP3 inflammasome inhibitors is anticipated to accelerate further.

#### 4.2.8 Application of New tricks for old drugs affecting NLRP3 Inflammasome

Drug development is a costly endeavor, both in terms of financial resources and time. Thus, discovering new therapeutic indications for existing drugs represents a viable and cost-effective strategy for treating uric acid metabolism disorders [67].

Dimethyl fumarate, primarily utilized for treating relapsing-remitting multiple sclerosis, has recently been shown to exhibit beneficial effects in alleviating inflammatory responses in rat models of acute gouty arthritis. This mechanism is attributed to its ability to inhibit the activation of NF-κB and NLRP3 inflammasomes, as well as the expression of caspase-1, IL-1β, and IL-18 [68]. These findings suggest that dimethyl fumarate could be a potential candidate for gout treatment.

Tranilast, an analogue of tryptophan metabolites, was initially approved for treating allergies and various inflammatory diseases. Recent research has revealed that tranilast can alleviate joint swelling and pain in mouse models of gouty arthritis by directly binding to the NACHT domain, disrupting NLRP3-NLRP3 oligomerization, and subsequently inhibiting NLRP3 inflammasome activation and IL-1β release [69]. Tranilast can also effectively inhibit urate reabsorption mediated by URAT1 and GLUT9, thereby reducing serum uric acid levels [70].

Moreover, drugs such as glyburide, simvastatin, and carboxyamidotriazole have demonstrated activity in inhibiting NLRP3 inflammasome activation, showing significant efficacy in conditions such as acute lung injury and inflammatory bowel disease [71, 72]. However, the potential role of these drugs in disorders related to uric acid metabolism remains largely unexplored. Consequently, repurposing existing drugs may be a promising option for future development in this field.

#### 4.2.8 Application of Synthetic Drugs Affecting the NLRP3 Inflammasome

Synthetic inhibitors of the NLRP3 inflammasome include sulfonylureas, sulfonamides, and urea derivatives [73]. MCC950, a sulfonylurea, stands out as a key NLRP3 inhibitor, serving as the basis for many synthetic NLRP3 inhibitors. MCC950 binds to the Walker B motif in NLRP3's NACHT domain, inhibiting ATP hydrolysis and locking NLRP3's active conformation, thus blocking inflammasome activation [74, 75]. However, Phase II clinical trials for MCC950 in rheumatoid arthritis were terminated due to elevated liver enzymes [76]. Despite this, MCC950 remains a crucial scaffold for designing NLRP3 inhibitors.

Other synthetic compounds have also been shown to inhibit NLRP3 inflammasome activation, primarily studied in models of inflammatory diseases such as pneumonia, colitis, and atherosclerosis. Further research into their potential for treating uric acid metabolism disorders is needed.

#### 4.2.9 Application of Natural Products in Modulating NLRP3 Inflammasome

The pursuit of natural, safe, and potent NLRP3 inflammasome inhibitors remains a pivotal focus in the prevention and treatment of uric acid metabolism disorders, as well as a vital pathway for novel drug discovery. Various natural products, such as flavonoids, alkaloids, terpenes, and plant extracts, have been shown to significantly inhibit NLRP3. Notably, the aqueous extract of Cyclocarya paliurus, abundant in flavonoids, effectively reduces XOD activity, modulates renal uric acid transporter proteins to decrease uric acid levels, and inhibits NLRP3 activation and IL-1β expression in the kidneys of hyperuricemic mice, thereby alleviating renal inflammation [77]. Additionally, nuciferine, a primary aporphine alkaloid derived from Nelumbo nucifera leaves, demonstrates therapeutic potential in treating hyperuricemia-induced renal inflammatory damage by lowering serum uric acid levels, reducing IL-1β secretion in systemic and renal tissues, and inhibiting NLRP3 inflammasome activation through the TLR4/MyD88/NF-κB signaling pathway [78].

#### 4.2.10 Application of Dietary Supplements in Modulating NLRP3 Inflammasome

In addition to medications, dietary supplements play a vital adjunctive role in managing uric acid metabolism disorders. These supplements are derived from natural food components, encompassing small-molecule phytochemicals like flavonoids and anthocyanins, as well as biological macromolecules like bioactive oligopeptides. Procyanidin B2, abundant in vegetables and fruits, effectively inhibits inflammatory responses by reducing the production of inflammatory factors [79]. The Hesperidin (Hsp)-Cu (II) complex significantly reduces blood uric acid levels and protects the kidneys by regulating uric acid metabolic enzymes and transporters, as well as alleviating renal oxidative stress [80]. Taxifolin, a natural flavonoid, reduces IL-1β production and enhances the phagocytic capacity of macrophages, thereby reducing inflammation in gout [81]. Furthermore, oligopeptides derived from tuna meat inhibit NLRP3 inflammasome activation, alleviating hyperuricemia-induced renal inflammation [82].

#### 4.2.11 Application of Chinese Herbal Medicines and Formulas Targeting NLRP3 Inflammasome

Chinese medicinal herbs and formulas play a pivotal role in preventing and treating uric acid metabolism disorders, owing to their multi-target, multi-pathway therapeutic effects and minimal adverse reactions. Herbs like chicory and gentian, along with formulas such as Simiao Powder and Wuling Powder, have been shown to significantly reduce uric acid levels, decrease urate deposition, inhibit inflammation, and effectively manage hyperuricemia, UN, and gout. For instance, chicory, a herb commonly used in Uyghur folk medicine, can significantly reduce serum uric acid levels by inhibiting uric acid production and enhancing uric acid excretion [83]. Additionally, chicory exhibits remarkable anti-inflammatory activity by inhibiting the secretion of inflammatory factors through the NF-κB/NLRP3 signaling pathway [84]. Furthermore, the classical formula Wuling San, derived from “Treatise on Febrile Diseases,” is frequently prescribed in clinical practice for treating hyperuricemia and its related nephropathies. Research has shown that Wuling San can effectively reduce IL-1β production in hyperuricemic mice by inhibiting TLR4/MyD88 signaling and NLRP3 inflammasome activation [85].

The extensive role of the NLRP3 inflammasome in uric acid metabolic disorders is recognized, but the safety of NLRP3-targeted drugs in preclinical studies and clinical trials remains a key concern, which requires further validation.

In preclinical studies, potential experimental methods should be used to further explore the mechanism of action of NLRP3 inhibitors and to comprehensively evaluate their safety. For example, using normal animals or animal models of hyperuricemia, uric acid nephropathy, and gouty arthritis, the efficacy and safety of NLRP3 inhibitors can be studied. Computer methods can be used to analyze the binding sites and modes of action between chemical components and NLRP3, optimize drug structure, improve drug specificity and affinity, and reduce side effects [100]. At the same time, research on the metabolism and excretion of NLRP3 inhibitors should be strengthened to reduce the accumulation of metabolites. Long-term safety assessment is crucial in clinical trials. For example, dapansutrile, a specific NLRP3 inflammasome inhibitor, demonstrated favorable safety and efficacy in alleviating joint pain among gout patients over a seven-day oral administration period in a Phase IIa trial [65]. However, its long-term safety profile must be rigorously evaluated through large-scale, multicenter clinical trials, particularly for chronic disease patients who require long-term medication. In addition to the experimental and clinical methods mentioned above, vigilant monitoring of drug side effects is crucial. Prolonged use of NLRP3 inflammasome inhibitors may induce toxic side effects, thereby limiting their clinical utility. For instance, MCC950, in a Phase II trial for rheumatoid arthritis, resulted in elevated serum liver enzyme levels, which reminds us that careful detection of drug side effects cannot be ignored [76]. Of course, personalized treatment should be considered due to patient variability [101]. Genomics and biomarkers can help tailor treatments, enhancing outcomes and safety [102]. Combining NLRP3 inhibitors with other drugs may better manage uric acid metabolic disorders and reduce side effects [103]. Future research will focus on ensuring the safety of NLRP3 drugs, aiming to develop safer, more effective treatments for uric acid metabolic disorders.

## CONCLUSION

This study employed systematic bibliometric analysis to reveal the evolving research hotspots related to the NLRP3 inflammasome in uric acid metabolism disorders from multiple dimensions. The findings highlight the rapid development of this field and provide new perspectives for future basic research and clinical applications. China and the United States have contributed substantially to the research output, with Chinese research institutions emerging as key players in advancing fundamental research. Initial research primarily focused on elucidating the molecular mechanisms of NLRP3 inflammasome activation and its pathological roles in gouty arthritis and uric acid nephropathy. As studies evolved, it became increasingly clear that the NLRP3 inflammasome also plays a crucial role in the pathogenesis of hyperuricemia. Furthermore, chemical compounds and drugs that target the NLRP3 inflammasome are gaining significant attention. This shift offers new avenues and approaches for early detection, therapeutic intervention, and drug development in managing uric acid metabolism disorders. However, it is imperative to prioritize drug safety and efficacy to safeguard patient health and enhance treatment outcomes. In conclusion, this study provides a theoretical reference for understanding the current status of the NLRP3 inflammasome in uric acid metabolism disorders.

## LIMITATIONS

This study has certain inherent limitations associated with bibliometric analysis methods. Firstly, the study only included English articles from the Web of Science database. Other databases, such as Scopus or CNKI, could yield additional valuable results. Secondly, the bibliometric analysis was conducted using multiple software tools, which may introduce variability in the results. Lastly, research is a dynamic and continuously evolving field, necessitating future updates to this analysis.

## Figures and Tables

**Fig. (2):**
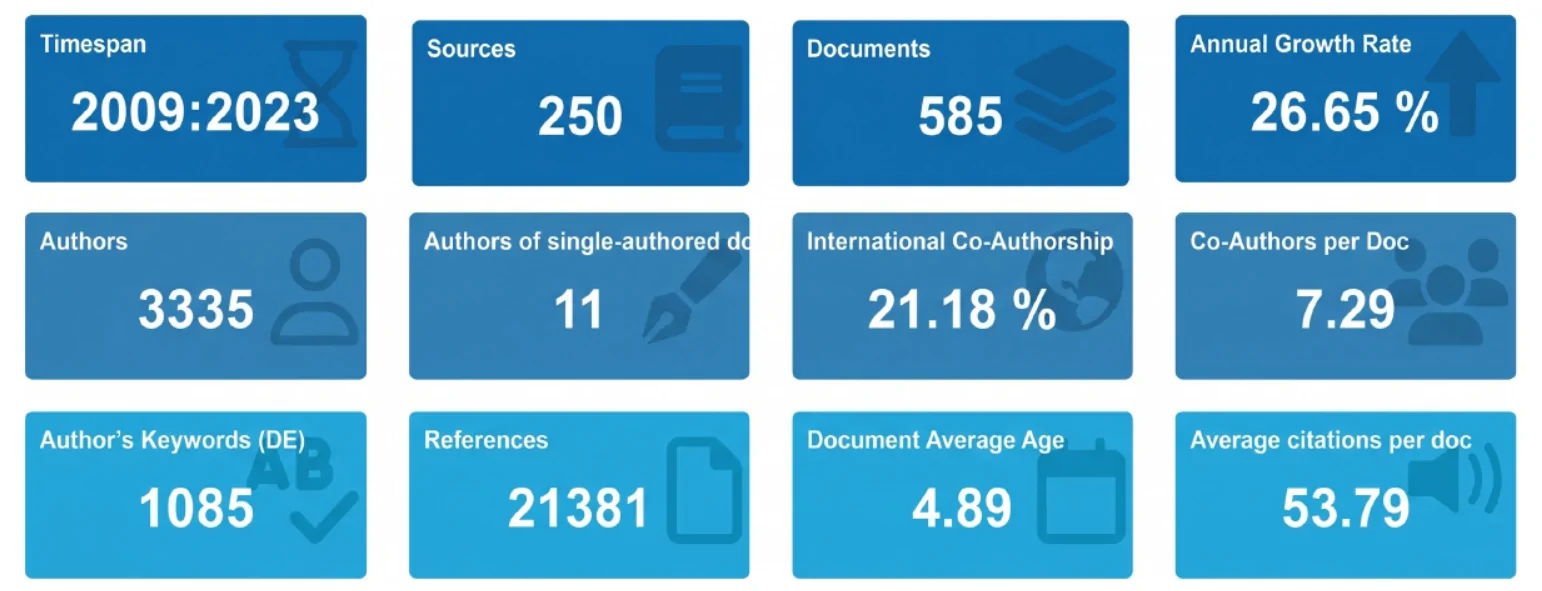
Overview of research on NLRP3 inflammasome and uric acid metabolism disorders (Data derived from R-bibliometric statistics).

**Fig. (3):**
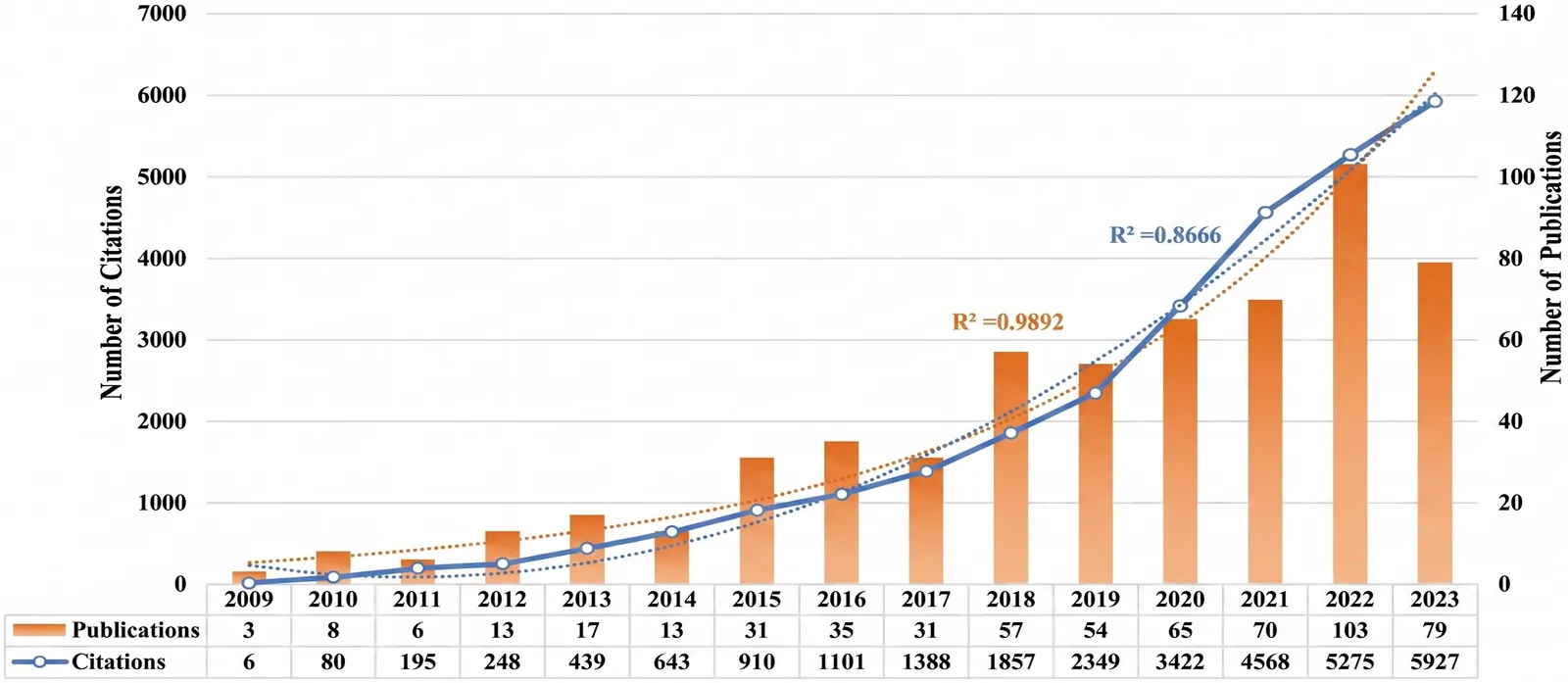
Number of publications and citation trends from 2009 to 2023. The orange nodes represent the number of publications, while the white nodes represent the number of citations. The orange dashed line represents the growth curve of publications, and the blue dashed line depicts the growth curve of citations.

**Fig. (5):**
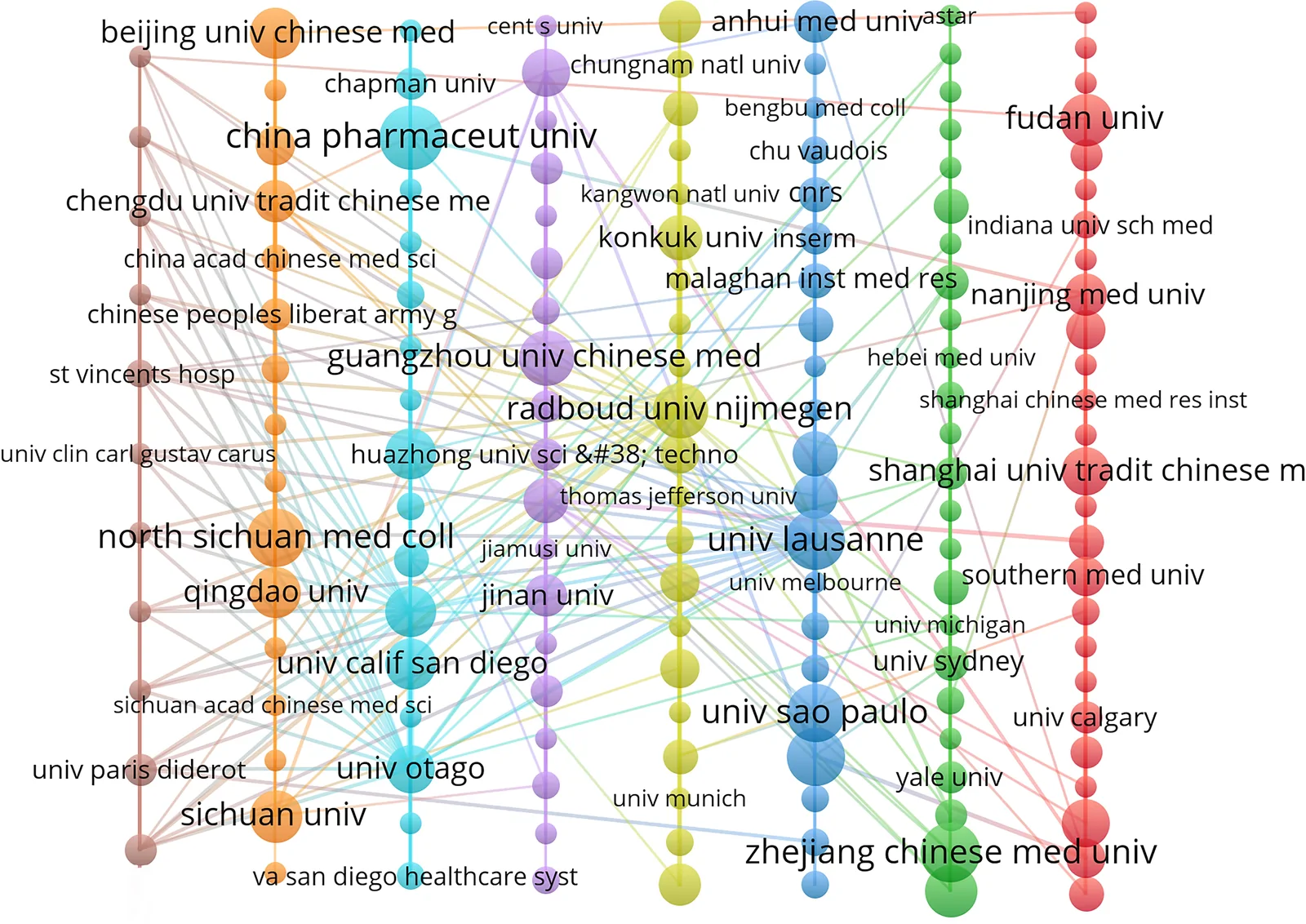
Graph of institutional collaboration clusters. Nodes represent institutions, links between nodes represent inter-institutional cooperation, node size represents the number of publications, and clusters of the same color indicate the presence of more active cooperation.

**Fig. (8):**
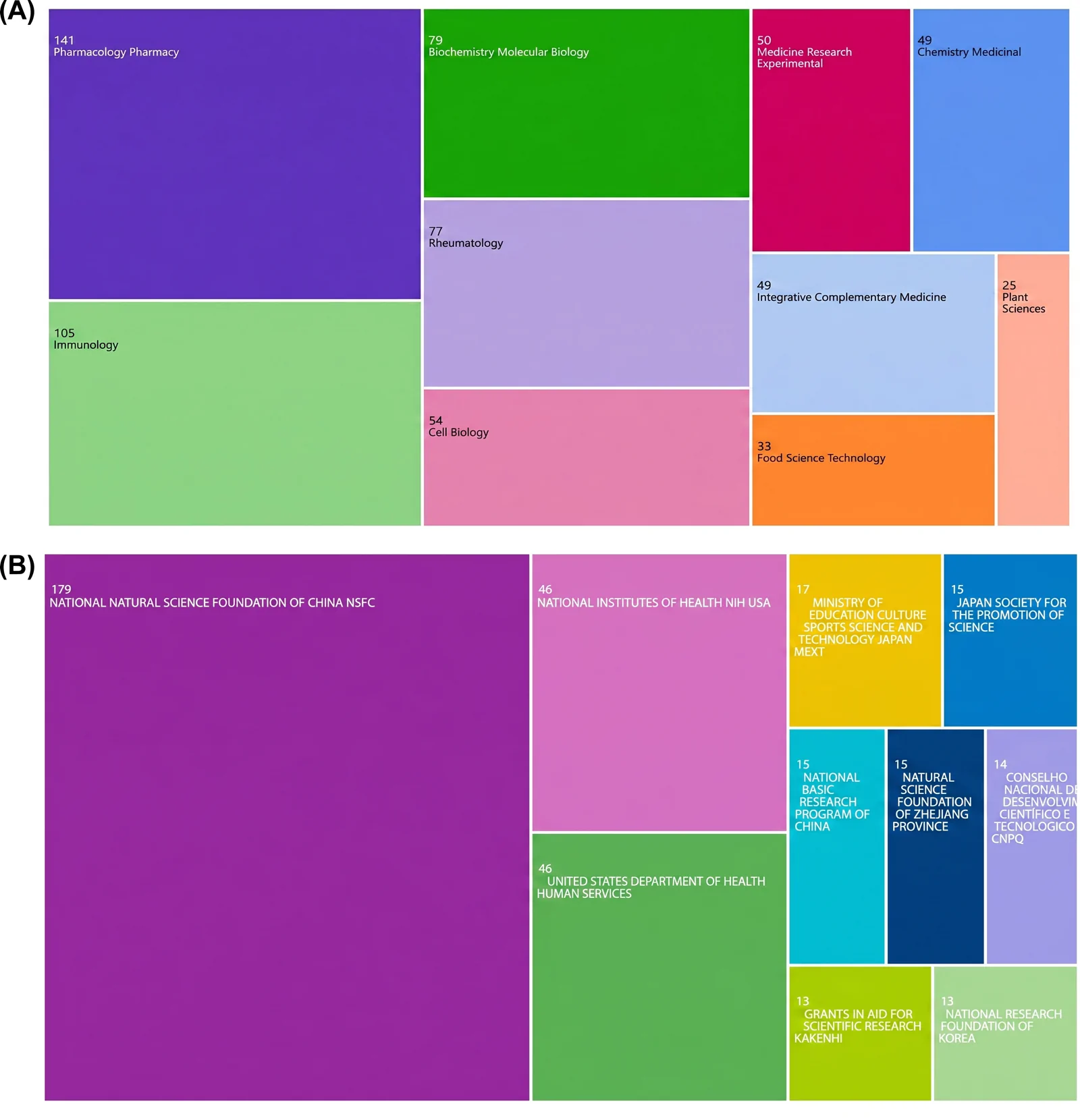
Analysis of publications by research category and funding agency. **(A)** Top ten research categories. **(B)** Top ten funding agencies.

**Fig. (9):**
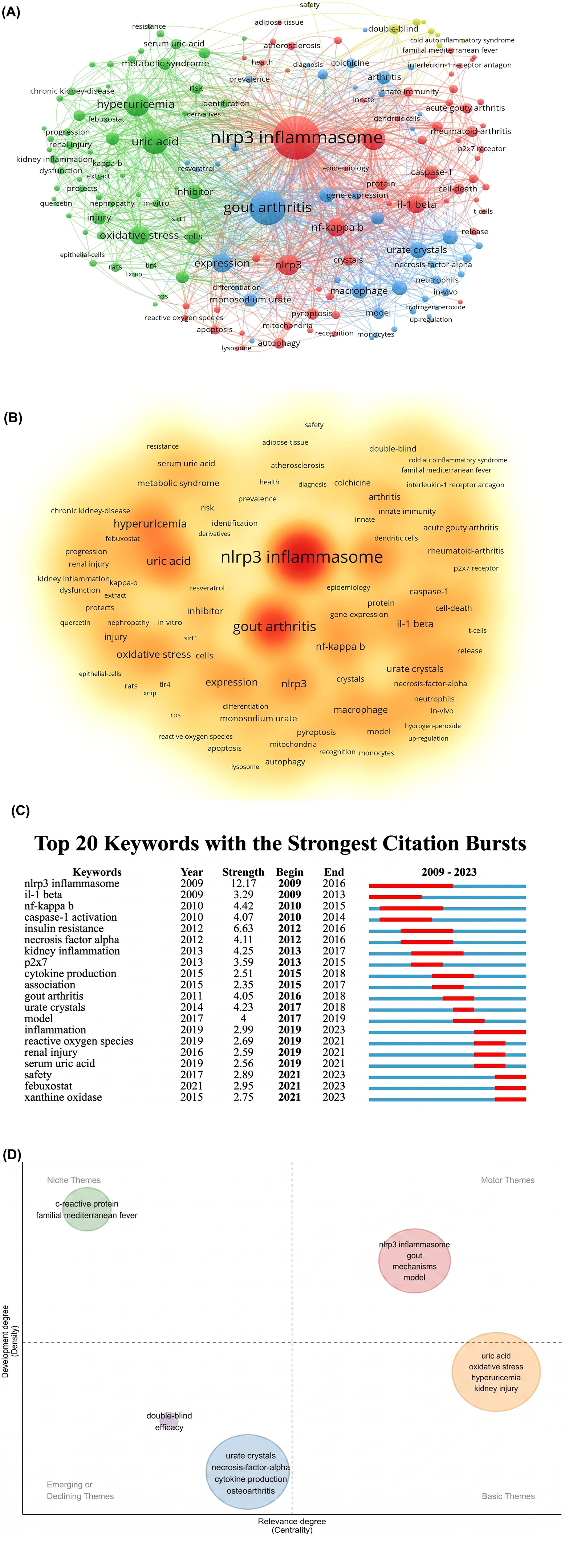
Keywords and thematic map analysis. **(A)** Keywords: cluster analysis. There are four categories, and the size of the nodes is proportional to the frequency of occurrence of the keywords. **(B)** Keywords: density analysis. The darker the color, the more concentrated the keyword research. **(C)** Keywords: burst analysis. The light blue color represents the period 2004-2023, the blue color represents the period of keyword research, and the red line represents the period of keyword research hotspots. **(D)** Topic words analysis. The x-axis indicates centrality; the larger the x, the stronger the centrality; the y-axis indicates density; and the larger the y, the more mature the study.

**Fig. (10):**
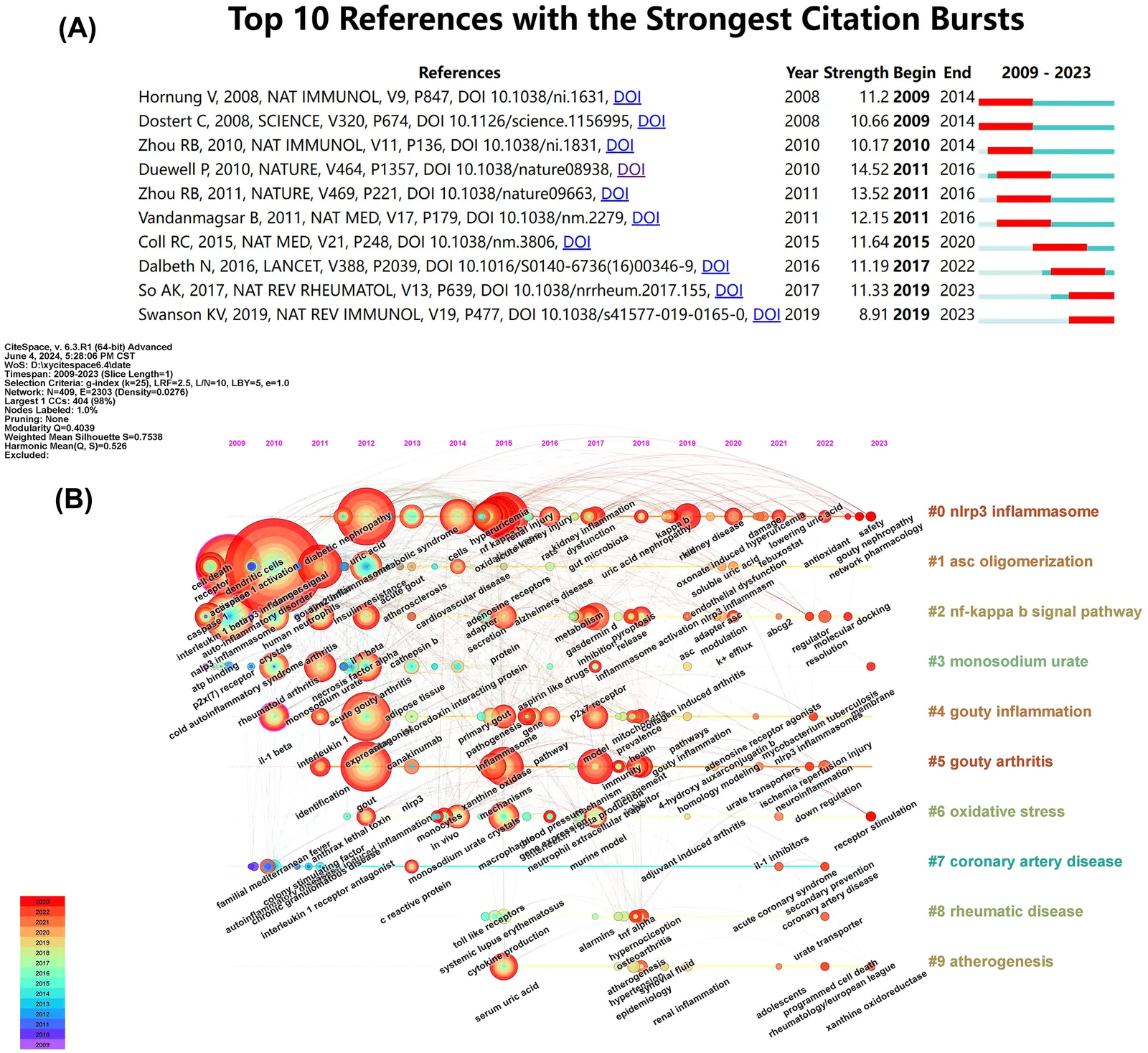
Co-citation analysis. **(A)** Top ten co-cited literature analyses. **(B)** Timeline distribution of keyword cluster analysis.

**Table 1:** Top 10 countries/regions with the most publications on NLRP3 inflammasome and uric acid metabolism disorders.

**Rank**	**Country/Regions**	**Publications**	**Rate (N/585) %**	**Citations**	**H-index**
1	China (Asia)	313	53.50	8713	46
2	USA (North America)	100	17.10	9331	47
3	South Korea (Asia)	33	5.64	1590	19
4	Germany (Europe)	26	4.44	2820	21
5	United Kingdom (Europe)	27	4.62	1096	17
6	Japan (Asia)	24	4.10	1766	15
7	Brazil (South America)	23	3.93	1182	15
8	Switzerland (Europe)	21	3.59	4028	19
9	Australia (Oceania)	18	3.08	1681	14
10	France (Europe)	18	3.08	982	15
10	Italy (Europe)	17	2.91	558	11

**Table 2:** The top ten most productive institutions.

**Rank**	**Institutions**	**Countries**	**Publications**	**Rate (N/585)%**
1	China Pharmaceutical University	China	16	2.74
2	Nanjing University	China	14	2.40
3	University of Lausanne	Switzerland	14	2.40
4	Zhejiang Chinese Medical University	China	14	2.40
5	North Sichuan Medical College	China	13	2.22
6	Universidade de São Paulo	Brazil	13	2.22
7	University of Science and Technology of China	China	13	2.22
8	Guangzhou University of Chinese Medicine	China	12	2.10
9	Fudan University	China	11	1.89

**Table 3:** Clinical trial drugs targeting NLRP3 inflammasome.

**Drug**	**Company**	**Structure**	**Clinical Phase**	**NCT Number** **/Latest Status**	**Information Sources**
**Dapansutrile** **(OLT1177^®^)**	Olatec	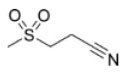	Phase II	NCT05658575 (Recruiting)	www.clinicaltrials.gov
/	Centre Hospitalier Saint Joseph Saint Luc de Lyon	Not disclosed	Not disclosed	NCT05744297 (Recruiting)	https://trialsearch.who.int/
/	Attune Health Research, Inc.	Not disclosed	Not disclosed	NCT04125459 (Not recruiting)	https://trialsearch.who.int/

**Table 4:** Representative NLRP3 inflammasome inhibitors.

**Type**	**Drug**	**Dose and Method of Administration**	**Pathway/Mechanism**	**Model/Object**	**References**
**New tricks of old drugs**	Dimethyl Fumarate	10 and 30 mg/kg/day, i.g.	Inhibition of ROS production and Caspase-1 activity	Gouty arthritis mouse model	[68]
	Tranilast	200 mg/kg, i.g.	Binds to the NACHT domain of NLRP3 to block NLRP3 oligomerization	Gouty arthritis mouse model	[69]
**Synthetic drugs**	MCC950	2 ml, 0.75 mg/ml, i.g.	Inhibition of NLRP3/IL1β signaling pathway	Gouty arthritis rat model	[86]
	New *N*-Sulfonylureas	3, 10 mg/kg, i.p.	reduce caspase-1 activation, oligomerization of ASC, and therefore, IL-1β processing	Gouty arthritis mouse model	[87]
	SLC3037	25 mg/kg, i.p.	Blocking the binding or oligomerization of NLRP3 and NEK7, as well as the oligomerization/phosphorylation of ASC	Gouty arthritis mouse model	[88]
**Natural products**	Cyclocarya paliurus aqueous extract	0.378, 1.140 g/kg, i. g.	blocking the activation of the ROS/NLRP3/IL-1β pathway	Hyperuricemia mouse model	[77]
	*Plantaginis Semen* polysaccharides	270 mg/ kg, i. g.	Downregulate the expression of NLRP3, ASC, and caspase-1 proteins in the kidneys and inhibit the release of IL-1β	Hyperuricemia nephropathy rat model	[89]
	Red ginseng extract	36 mg/day, o.p.	Inhibit ASC oligomerization and block NLRP3 inflammasome assembly	Gout patients	[90]
	*Actinidia arguta* extract	25, 50 mg/kg, i. g.	Regulating NLRP3 ubiquitination and ASC oligomerization, inhibiting caspase-1 activation	MSU-induced peritonitis mouse model	[91]
	Isoorientin	5, 10 mg/kg, i. g.	Regulating the TLR4/NLRP3 signaling pathway	Hyperuricemia rat model	[92]
	Nuciferine	10, 20, and 40 mg/kg, i. g.	Inhibition of TLR4/MyD88/NF - κB signaling pathway and NLRP3 inflammasome activation	Hyperuricemia mouse model	[78]
	Emodinol	56, 112 mg/kg, i. g.	Inhibition of NLRP3, ASC, and Caspase-1 expression, inhibition of TLRs/MyD88/NF - κB signaling pathway	Hyperuricemia nephropathy rat model	[93]
	Rutin	150, 300 mg/kg, i. g.	Inhibit ROS production, restore oxidative stress balance, and inhibit NLRP3 inflammasome activation	Gout quail model	[62]
**Dietary supplement**	Procyanidin B2	60 mg/kg, i. g.	Inhibition of NLRP3 expression and IL-1β release	Mouse airbag model and gouty arthritis model	[79]
	Hesperitin-Copper(II) Complex	30, 60, and 120 mg/kg, i. g.	Inhibition of NLRP3, ASC, and caspase-1 expression	Hyperuricemia mouse model	[80]
	Taxifolin	100, 200 mg / kg, i. g.	Inhibit Caspase-1 expression and reduce IL-1β release	Gouty arthritis rat model	[81]
	Tuna meat oligopeptides	50, 300 mg / kg, i. g.	Inhibition of NLRP3 inflammasome and activation of TLR4/MyD88/NF - κB signaling pathway, inhibition of p65 NF - κB phosphorylation	Hyperuricemia mouse model	[82]
	Rice-derived-peptide-2	5, 10, and 100 μg / kg, i.p.	Inhibit the expression of NLRP3, Caspase 1, ASC, and excessive production of IL-1β	Gouty arthritis rat model with hyperuricemia	[94]
**Traditional Chinese medicine and prescriptions**	*Cichorium intybus* L.	7.5, 15 g / kg, i. g.	Inhibit NF kappa B and NLRP3 signaling pathways and reduce IL-1β release	Gouty arthritis rat model	[83]
	Euodiae fructus	0.375, 0.75 g / kg, i. g.	suppresses NLRP3-mediated ASC oligomerization	Gout mouse model with high blood uric acid	[95]
	*Cinnamomum cassia*	25 mg/kg, i. g.	Reduce the production of IL-1β	Gouty arthritis mouse model	[96]
	Simiao pill	8.87, 157.74 mg / kg, i. g.	Inhibition of NF - κ B/NLRP3 signaling pathway	Hyperuricemia-induced renal injury model in rats	[97]
	Simiao San	4.55, 9.1, 18.2 g / kg, i. g.	Reduce the expression of NLRP3, ASC, caspase-1, and JAK2/STAT3 signaling pathways	Hyperuricemia-induced renal injury model in rats	[98]
	Fuling-Zexie formula	500 mg/kg, i. g.	Inhibition of JAK2/STAT3 signaling pathway and NLRP3 inflammasome activation	Hyperuricemia rat model	[99]
	Wuling San	987, 1316, 1755 and 2340 mg/kg, i. g.	Inhibition of TLR4/MyD88 signaling pathway and NLRP3 inflammasome activation, reducing IL-1 β secretion	Hyperuricemia mouse model	[85]

**Note:** The safety of NLRP3 inflammasome-related drugs may emerge as a future research trend.

## LIST OF ABBREVIATIONS

NEK7: NIMA-Related Kinase 7
NSFC: National Natural Science Foundation of China
UN: Uric Acid Nephropathy
Woscc: Web of Science Core Collection
